# Age-Related Human Adaptation to Extreme Climatic Factors and Environmental Conditions

**DOI:** 10.3390/biology14121668

**Published:** 2025-11-25

**Authors:** Anna E. Kallio, Ekaterina A. Davydova, Tatiana A. Mishchenko, Tatiana M. Sivtseva, Maria M. Nikolaeva, Dmitriy E. Burmistrov, Anzhela D. Bolshakova, Sergey N. Tsybusov, Raisa N. Zakharova, Maria V. Vedunova

**Affiliations:** 1 Institute of Biology and Biomedicine, Lobachevsky State University of Nizhny Novgorod, 603022 Nizhny Novgorod, Russia; ankallio26@gmail.com (A.E.K.); spring_dusk@mail.ru (E.A.D.); saharnova87@mail.ru (T.A.M.); anzhela.bolschakova@yandex.ru (A.D.B.); tzibusov56@mail.ru (S.N.T.); 2 Research Center of the Medical Institute, M.K. Ammosov North-Eastern Federal University, 677013 Yakutsk, Russia; tm.sivtseva@s-vfu.ru (T.M.S.); mariaokhotina@mail.ru (M.M.N.); prn.inst@mail.ru (R.N.Z.); 3 Prokhorov General Physics Institute of the Russian Academy of Sciences, 119991 Moscow, Russia; dmitriiburmistroff@gmail.com

**Keywords:** age acceleration, biological age, biochemical analysis, clinical blood parameters, cold environment, urbanization

## Abstract

The exposome, which encompasses lifetime environmental exposures, significantly impacts health and can act as a major driving force in accelerating the aging process. While urbanization and climate are among the key exposome factors, the mechanisms that facilitate physiological adaptation to their effects and could potentially extend longevity remain poorly understood. In this study, we aimed to identify aging-associated biomarkers influenced by environmental exposures by comparing hematological and biochemical parameters from 445 individuals. The cohort comprised residents of an extremely cold climate region and of several cities with a milder climate, representing varying levels of urbanization. Our findings, obtained using the Levine PhenoAge model and machine learning, indicate that living in extreme climatic conditions promotes age acceleration, which is accompanied by decreased blood albumin levels. We also observed a strong positive correlation between age acceleration and the functional parameter of erythrocytes (RDW-CV) across different climatic zones. Within the temperate climate zone, residents of small towns exhibited the slowest age acceleration, highlighting urbanization as a driver of accelerated aging. Our data enhance the understanding of the role of the exposome in aging and could inform strategies for promoting healthy aging and longevity tailored to specific environmental and urban contexts.

## Simple Summary

Despite the easy availability of modern medical technologies, life in a big city—with its heavy traffic and numerous industries—is not always beneficial for our health. Meanwhile, there are regions on our planet where life seems extremely challenging due to harsh environmental conditions. Nevertheless, human settlements exist even in places where winter temperatures can drop to −70 °C. How is this even possible? How do people live in such extremes, and is it possible to live a long and healthy life there? In our study, we assessed the biological age of individuals from two distinct regions: one known as the “cold pole of the planet” and the other, a central region with a milder climate. The participants from the central region included residents of a metropolis, an industrial city, and two small towns. We compared not only the rate of biological aging but also the specific indicators responsible for age-dependent adaptation to the conditions of the Far North. We identified patterns in blood parameters associated with the rate of aging, which showed distinct regional variations. Differences between residents of large and small cities were also observed.

## 1. Introduction

Human aging is a complex, multifactorial process characterized by progressive physiological deterioration, increased susceptibility to age-related diseases, and an inevitable decline in functional capacity [1]. The search for the causes of aging and ways to delay the onset of age-related pathologies remains a central and highly demanding goal in geroscience [2].

The entirety of external and internal factors that significantly impact health and can act as a major driving force in accelerating the aging process are currently encompassed by the term “exposome” [3,4]. Recent evidence underscores the critical role of environmental toxicants, such as particulate matter, pesticides, heavy metals, and organic solvents, in accelerating aging processes and shaping the rate of cardiovascular and brain pathologies [5,6]. Alongside the effects of urbanization, the climate itself can act as a significant factor influencing human health and life expectancy [7]. For instance, prolonged exposure to extreme temperatures, both hot and cold, can impair cardiovascular function, thereby increasing the risk of myocardial infarction, malignant cardiac arrhythmias, thromboembolic disease, and heat-induced sepsis [8].

A limited number of studies have shown that indigenous populations inhabiting cold climates, such as the Far North of Russia, develop genetic adaptations that enhance survival in severe cold conditions [9]. Empirical studies have shown that residents of Eastern Siberia exhibit lower serum lipid levels [10] and higher blood pressure [11,12] compared to populations in other geographic regions. Furthermore, comprehensive genomic studies of Siberian populations have identified candidate genes associated with cold adaptation, energy regulation, and metabolism, which are essential for maintaining homeostasis in extreme environments [9]. Moreover, extreme climatic conditions have been shown to affect epigenetic markers [13,14]. Available data also suggests that epigenetic aging occurs at a significantly accelerated rate in the Yakutian population compared to that in Central Russia [15].

It is important to note that the contribution of the exposome to accelerated aging remains poorly characterized and requires further in-depth investigation. In particular, a notable gap exists in the current data on biological age profiles of populations residing in extreme climates.

Biological age determination based on blood chemistry data analysis using modern machine learning methods allows for the extraction of complex patterns from large datasets. In this study, using the Levine PhenoAge model and machine learning, we aimed to compare hematological and biochemical parameters between residents of an extreme cold climate (the Republic of Sakha (Yakutia)) and those from the milder climate of Central Russia (the Nizhny Novgorod region) to identify aging-associated markers linked to environmental exposures. We also compared the analyzed blood parameters and assessed the level of accelerated aging for residents of Central Russia from various urbanized territories, including a metropolis (Nizhny Novgorod), an industrial city (Dzerzhinsk), and two small towns with a population of less than 50,000 people (Semenov and Pavlovo). Our approach significantly expands our understanding of various aspects of the aging process, including the features of physiological adaptation to the impact of the exposome, particularly to extreme factors, and the maintenance of healthy longevity under such conditions.

## 2. Materials and Methods

### 2.1. Data Collection and Cohort Characterization

**Figure 1:**
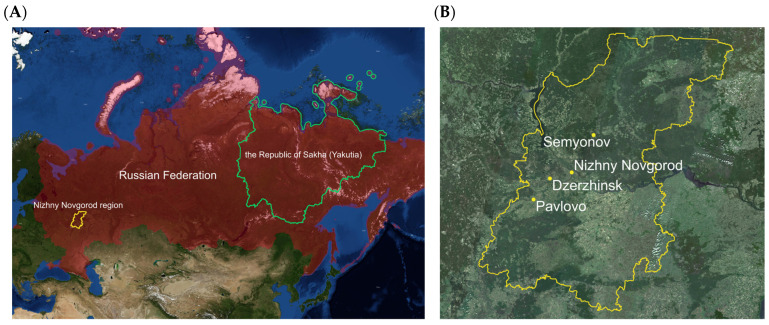
Regions of participant recruitment. (**A**) Overview within the territory of the Russian Federation: Yakutia (the Republic of Sakha, highlighted in green) and the Nizhny Novgorod region in Central Russia (highlighted in yellow); (**B**) Study sites within the Nizhny Novgorod region: the metropolis of Nizhny Novgorod, the industrial city of Dzerzhinsk, and the two small towns of Semenov and Pavlovo, each with a population under 50,000.

This study utilized data from hematological and biochemical tests of 445 volunteers from two distant geographic regions—the Republic of Sakha (Yakutia, highlighted in green in Figure 1) and the cities within the Nizhny Novgorod region in Central Russia (highlighted in yellow in Figure 1) with varying levels of urbanization.

The population of Yakutia endures extreme environmental conditions, including the prolonged severe cold (with winters lasting from October to April, averaging −42 °C), substantial seasonal fluctuations in daylight hours, and limited access to fresh vegetables, fruits, and greens. In contrast, the Nizhny Novgorod region has a relatively milder climate, characterized by higher average temperatures and a shorter winter (with the period of negative temperatures lasting from November to March and an average winter temperature of −13 °C). The populations selected for our analysis also exhibit genetic differences: the inhabitants of Central Russia share genetic affinities with the peoples of Northeastern Europe, whereas the genetic composition of the Yakutian population reflects characteristics typical of Siberian and East Asian groups [15].

All study participants were self-reported as healthy. Exclusion criteria included chronic diseases in an acute stage, cancer, acute respiratory viral infections, or pregnancy at the time of blood sample donation. The study was conducted in accordance with the ethical principles of medical research involving human subjects as outlined in the 9th revision of the Declaration of Helsinki (World Medical Association, October 2013). The study protocol was approved by the Local Ethics Committee of Lobachevsky University (Protocol No. 1, 2 December 2020). All participants provided written informed consent. Blood samples were collected from study participants between 2020 and 2023.

A total of 445 study participants were categorized into four groups based on their environmental and climatic profile:

(1) Yakutia—individuals residing in the extreme climatic conditions of the Republic of Sakha (Yakutia), in small settlements with a moderate level of anthropogenic impact (n = 153);
(2) Nizhny Novgorod—volunteers living in the large metropolis of Nizhny Novgorod in Central Russia, which has a temperate continental climate featuring higher average temperatures and shorter winter period (n = 144);
(3) Dzerzhinsk—residents of the industrial city of Dzerzhinsk in the Nizhny Novgorod region, characterized by elevated levels of air, water, and soil pollution (n = 60);
(4) Small towns—volunteers living in the small towns Semyonov and Pavlovo in the Nizhny Novgorod region, with populations of up to 50,000 (n = 88).

A detailed description of the ecological characteristics for each studied region is available in the Supplementary Materials.

**Figure 2:**
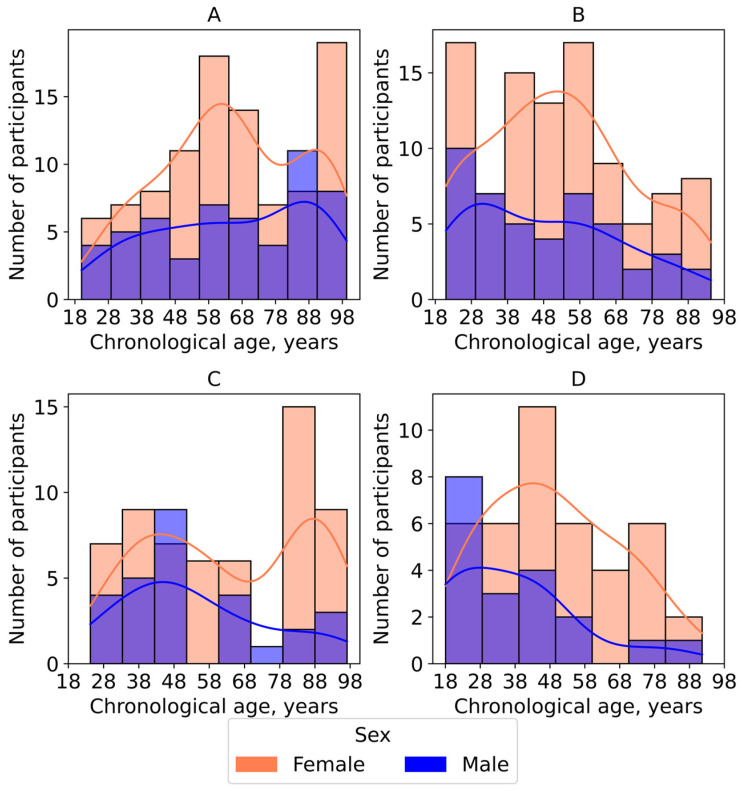
Density plots representing the age and sex distribution in the study groups of volunteers. (**A**) Yakutia; (**B**) Nizhny Novgorod; (**C**) Small towns; (**D**) Dzerzhinsk.

The age range of participants is shown in Figure 2. In all study groups, the number of female participants exceeded that of male volunteers (Figure 2).

### 2.2. Sample Handling

To ensure standardization, blood collection and subsequent sample processing were performed according to a protocol strictly followed by the laboratories in the study regions. Blood samples were collected in the morning after an 8–12 h fast. Blood sample collection and analysis of general clinical blood analysis of participants from Nizhny Novgorod, Dzerzhinsk, Semyonov, and Pavlovo was performed at the central district hospitals in each respective city/town. The volunteers from Yakutia were indigenous individuals from Yakutsk and nearby rural municipalities (uluses).

Blood samples were collected in VACUETTE vacuum tubes (Greiner Bio-One, Kremsmünster, Austria) containing K3-EDTA (for hematological tests) or lithium heparin (for biochemical tests). Plasma was obtained by centrifuging the blood samples at 3000 rpm for 10 min. The samples were subsequently stored at −80 °C. According to the protocol, the time from blood draw to freezing did not exceed two hours.

### 2.3. Analytical Methods

Fresh whole blood samples were analyzed to determine 18 general parameters using the Mindray BC-20s hematology analyzer (Mindray, Shenzhen, China) and biochemical parameters using an automatic biochemical analyzer DIRUI CS-T240 (Dirui, Changchun, China) (Table 1).

Quality control for the hematological and biochemical analyses was performed using Gematrol 3D (Medical and Biological Union, Novosibirsk, Russia) and Trulab P and N (DiaSys, Holzheim, Germany), respectively. Quality control results were within certified ranges.

### 2.4. Biological Age Calculation

The biological age of the study participants was calculated using the PhenoAge model [16]. This model is widely used in aging research and clinical studies as it reflects the cumulative physiological burden and overall health status, enabling early identification of accelerated aging phenotypes and informing potential interventions. The PhenoAge model is based on chronological age and nine clinical and biochemical blood parameters, including WBC, MCV, LYM (%), RDW-CV (%), albumin, glucose, creatinine, ALP, and CRP.

The linear predictor (w) was constructed as: −0.0336 × albumin (g/L) + 0.0095 × creatinine (umol/L) + 0.1953 × glucose (mmol/L) + 0.0954 × log_10_(CRP (mg/L) × 10) − 0.0120 × lymphocyte (%) + 0.0268 × MCV (fL) + 0.3306 × RDW-CV (%) + 0.0019 × ALP (U/L) + 0.0554 × WBC (10^9^/L) + 0.0804 × age (years) − 19.9067. The computed linear score w was then transformed into biological age using the original published formula [16]. All formula constants, exponential, and logarithmic terms are retained exactly as in the original publication [16].

### 2.5. Data Processing

All analyses were performed in Python 3.12.4, utilizing standard libraries for data manipulation (Pandas 2.2.2, NumPy 1.26.4), machine learning (Scikit-learn 1.4.2), statistics (scipy.stats subpackage, SciPy 1.13.1), and visualization (Matplotlib 3.8.4, Seaborn 0.13.2.).

Biological age acceleration is defined as the deviation of estimated biological age from chronological age and is typically measured either as the difference between biological and chronological age or as the residual from the regression of biological age on chronological age [17]. In this study, age acceleration was calculated as the difference between biological age and the predicted age derived from a general regression model constructed for all study groups.

Statistical significance of age acceleration across study groups was assessed using the Mann–Whitney test with the Benjamini–Hochberg correction.

The relationship between blood parameters and age within the study groups was visualized using a Gaussian weighted moving average using the Gaussian_filter1d function from the Ndimage submodule of SciPy.

### 2.6. Machine Learning

Machine learning (ML) approaches were employed to investigate age-related changes in volunteers residing in different climatic zones. All data were normalized using the AdjustedScaler class from the AdjDataTools library (available at https://github.com/newchronik/adjdatatools (accessed on 10 June 2025)), as both principal component analysis and K-means clustering are sensitive to data scaling. The AdjustedScaler is a robust preprocessing tool that uses the medcouple statistic to handle skewed data and outliers [18], addressing limitations of traditional scalers like StandardScaler or MinMaxScaler.

In this study, principal component analysis was conducted using the sklearn.decomposition module. To determine the optimal number of components, a graph was constructed to illustrate the increase in total variance with an increasing number of components, and the “elbow test” was applied [19]. Clustering was performed using the KMeans class from the sklearn.cluster module on standardized data after dimensionality reduction via PCA. Centroids were initialized using the k-means++ method, and the dataset was partitioned into three clusters.

## 3. Results

### 3.1. Age Acceleration Rate Across the Studied Groups

**Figure 3:**
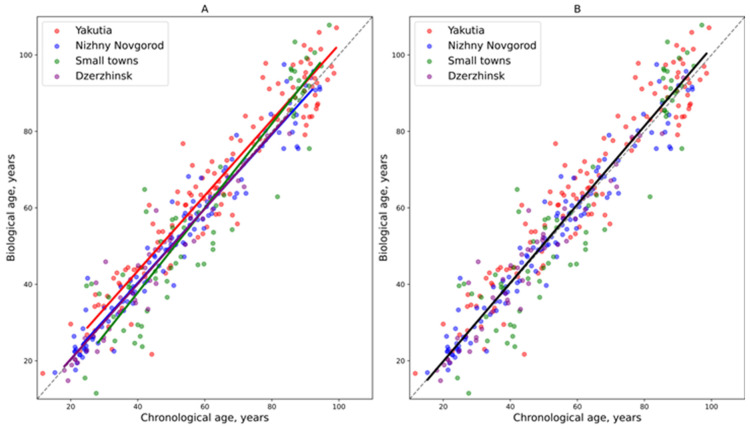
Linear regression models depicting the relationship between biological and chronological age in the study groups of residents. (**A**) Separate models for each study group. (**B**) General regression model. The dashed line indicates the point at which biological and chronological ages are equal.

According to the regression models, residents of the metropolis of Nizhny Novgorod and the industrial city of Dzerzhinsk exhibited similar aging rates, with their biological age being approximately equal to their chronological age on average (Figure 3). In contrast, among the indigenous population of Yakutia, biological age was elevated relative to chronological age. Participants from small towns of Semyonov and Pavlovo in the Nizhny Novgorod region demonstrated lower biological age values at younger ages. However, their rate of aging increased over time, resulting in a biological age that surpassed their chronological age.

**Figure 4:**
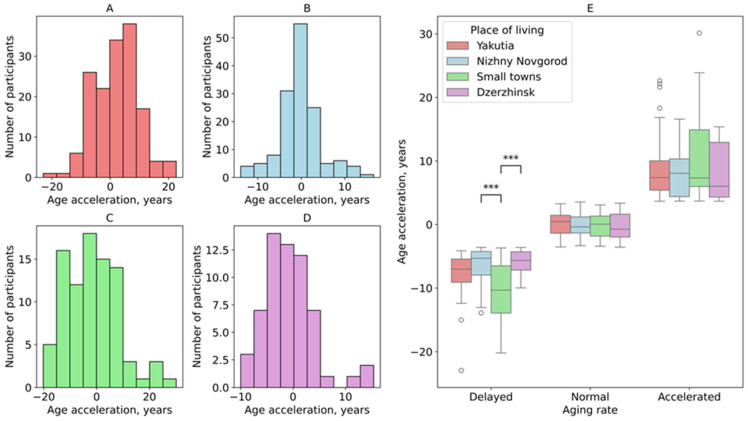
Histograms of age acceleration distribution in the study groups of residents. (**A**) Yakutia; (**B**) Nizhny Novgorod; (**C**) Small towns; (**D**) Dzerzhinsk; (**E**) Biological age acceleration across four study locations, grouped by aging rate. ***: 0.0001 < *p* ≤ 0.001.

Biological age acceleration was calculated for each study group. The histograms in Figure 4 revealed that the residents of Yakutia were characterized by predominantly higher values of age acceleration (Figure 4A). Differences in biological age acceleration between Yakutia and the metropolis of Nizhny Novgorod (effect size (rbc) = −0.224, 95% CI: −0.126, 0.138, *p* = 0.00084), Yakutia and small towns in the Nizhny Novgorod region (effect size (rbc) = −0.265, 95% CI: −0.154, 0.140, *p* = 0.00062), and Yakutia and the industrial city of Dzerzhinsk (effect size (rbc) = −0.267, 95% CI: −0.167, 0.162, *p* = 0.00245) were statistically significant, which aligns with the existing literature [15].

The residents of Nizhny Novgorod exhibited a normal rate of aging, with most age acceleration values near zero (Figure 4B). In contrast, participants from small towns in the Nizhny Novgorod region (Figure 4D) and the industrial city of Dzerzhinsk (Figure 4C) exhibited lower rates of biological age acceleration, with the slowest aging process evident in small-town residents. However, no significant differences in age acceleration were found among the cities within the Nizhny Novgorod region, likely due to their geographic proximity.

Based on the obtained biological age acceleration values, study participants were categorized into three groups: normal aging (±0.5 SD from the predicted biological age), delayed aging (below this range), and accelerated aging (above this range) (Figure 4E). Over half of the participants in Nizhny Novgorod and Dzerzhinsk showed a normal aging rate, with few cases of accelerated aging (15% and 13%, respectively). In contrast, the residents of Yakutia had a three times higher rate of accelerated aging (44%) than in the aforementioned cities. Small towns in the Nizhny Novgorod region had twice as many slow-aging individuals than in Yakutia and Nizhny Novgorod (Supplementary Table S1). Within the slow-aging group, significant differences were observed between the small towns and the industrial city of Dzerzhinsk (effect size (rbc) = −0.497, 95% CI: −0.279, 0.277, *p*-value = 0.0002), as well as the metropolis of Nizhny Novgorod (effect size (rbc) = 0.607, 95% CI: −0.326, 0.323, *p*-value = 0.0004).

### 3.2. Analysis of Regional Age-Related Variations in Blood Parameters

**Figure 5:**
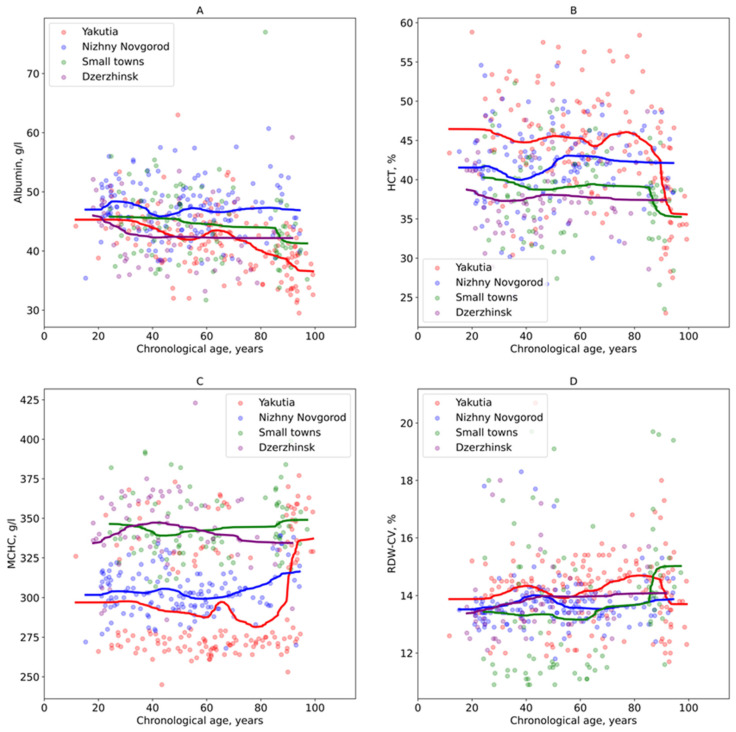
Dependence of selected clinical blood parameters on chronological age in the study groups of residents. (**A**) Albumin; (**B**) Hematocrit (HCT); (**C**) Mean corpuscular hemoglobin concentration (MCHC); (**D**) Platelet distribution width by volume (PDW-CV). The color lines mean Gaussian moving average of parameters in the study groups.

An assessment of age-related changes in key clinical and biochemical blood parameters revealed that albumin levels remained relatively stable with age among residents of Nizhny Novgorod and the industrial city of Dzerzhinsk (Figure 5A). On the other hand, the albumin levels in residents of Yakutia began to decline after the age of 35, while a decrease was observed only after 80 years of age in residents of the small towns in the Nizhny Novgorod region (Figure 5A).

After 80 years, a sharp decline in HCT in participants from Yakutia and small towns in the Nizhny Novgorod region was also shown (Figure 5B). Notably, before this decline, Yakutia residents had higher HCT levels than those from the Nizhny Novgorod region.

Participants from small towns in the Nizhny Novgorod region and the industrial city of Dzerzhinsk demonstrated higher overall MCHC levels (Figure 5C). In contrast, MCHC values in residents of the metropolis of Nizhny Novgorod began to increase only after the age of 60. Interestingly, the Yakut population generally had lower MCHC levels compared to the other groups, but experienced a sharp increase after the age of 80.

Although the differences in RDW-CV were less pronounced, a generally higher trend was observed in Yakutia residents compared to participants from the Nizhny Novgorod region (Figure 5D). After 80 years, however, this trend reversed, with levels decreasing in Yakutia and increasing in Nizhny Novgorod region.

Age-related dependencies for the other studied blood parameters are shown in Supplementary Figure S1.

### 3.3. Principal Component Analysis and Clustering

The conducted principal component analysis allowed to reduce the dataset from 18 variables (RBC, WBC, HGB, MCV, HCT, MCH, MCHC, RDW-CV, PLT, LYM, albumin, glucose, creatinine, ALP, CRP, chronological age, biological age, and age acceleration) to 12 components, explaining 95% of the total variance. The first principal component explained 18.6% of the variance, the second explained 15.7%, and the third explained 12.5% (cumulative variance 46.8%). A constructed correlation matrix is presented in Supplementary Figure S2. The first principal component demonstrated the highest correlation with biological age and chronological age (r = 0.91 and 0.81, respectively). The second component was significantly associated with MCHC (r = −0.77), HCT (r = 0.75), RBC (r = 0.66), and age acceleration (r = 0.6). The third component exhibited stronger correlations with HGB (r = −0.84), HCT (r = −0.58), and MCH (r = −0.56).

**Figure 6:**
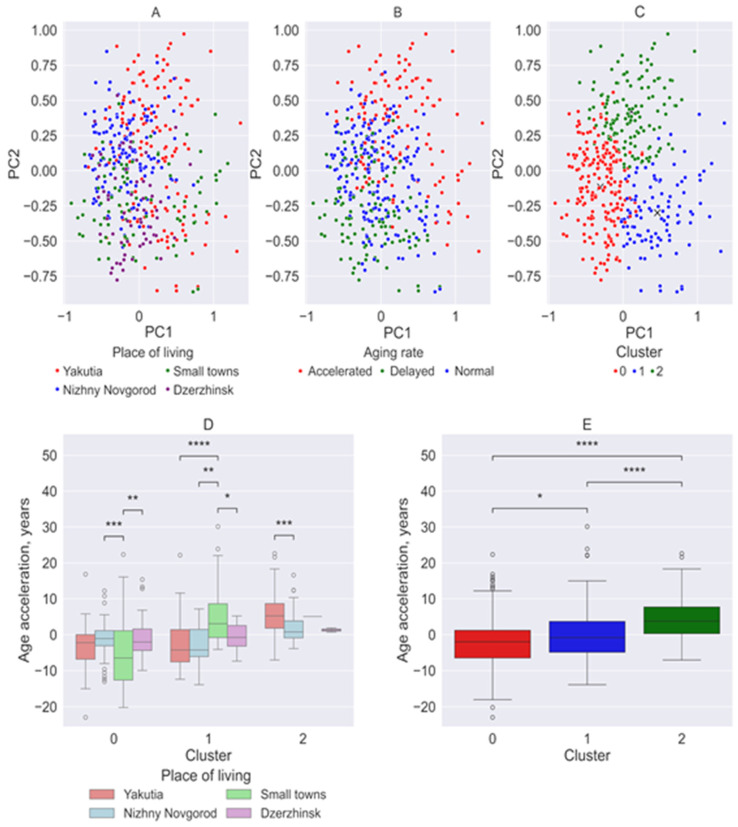
Principal component analysis and clustering. (**A**) Scatter plots of the first two principal components divided by place of residence. (**B**) Scatter plots of the first two principal components divided by aging rate. (**C**) Visualization of clusters in the coordinates of the first two components. (**D**) Graphs of biological age acceleration grouped by place of residence. (**E**) Graphs of biological age acceleration between clusters. *: 0.01 < *p* ≤ 0.05, **: 0.001 < *p* ≤ 0.01, ***: 0.0001 < *p* ≤ 0.001, ****: *p* ≤ 0.0001. The "×" symbols on the graph represent the cluster centroids.

The first two components, which accounted for the majority of the variance (34.3%), were selected for scatter plot visualization (Figure 6A). The data points that residents from the small towns of the Nizhny Novgorod region exhibited negative values for the second component, while participants form Yakutia predominantly had positive values for the first component. The scatter plots for observations from Nizhny Novgorod and Dzerzhinsk showed similar distributions, although the residents of Dzerzhinsk had more negative values for the second component.

When divided into groups based on the rate of aging (Figure 6B), as observed with classification by place of residence, the individual groups do not form distinct clusters but rather exhibit considerable overlap. Nevertheless, a general trend is evident: participants in the slow-aging group tend to have more negative values for both components, whereas individuals in the accelerated-aging group show more positive values.

The clustering analysis failed to distinguish groups by aging rate or place of residence, indicating that despite significant differences among study groups, a clear classification based solely on the analyzed parameters remains challenging (Figure 6C).

Despite the limitations in clustering accuracy, certain trends were identified (Figure 6C). Cluster 0 (red) predominantly represented the Nizhny Novgorod region, including the cities of Nizhny Novgorod, Dzerzhinsk, and to a lesser extent, the towns of Semyonov and Pavlovo). Cluster 2 (green) primarily consisted of participants from Yakutia. Cluster 1 (blue) contained a mix of participants from both Yakutia and small towns in the Nizhny Novgorod region, suggesting a potential link to their distance from major urban centers.

The resulting clusters exhibited statistically significant differences in biological age acceleration (Cluster 0 vs. 1, *p* = 0.012; Cluster 0 vs. 2, *p* = 0.00000001; Cluster 1 vs. 2, *p* = 0.000000001) (Figure 6E).

Cluster 0 is also characterized by lower biological age acceleration (Figure 6D). Residents of small towns in the Nizhny Novgorod region within this cluster showed significantly different values compared to residents of Nizhny Novgorod (*p* = 0.0002) and Dzerzhinsk (*p* = 0.003). Cluster 1 is characterized by accelerated aging in small towns in the Nizhny Novgorod region, whereas residents of Yakutia and Nizhny Novgorod exhibit slower aging (Figure 6D). Cluster 2, which is associated with higher biological age acceleration, revealed statistically significant differences between residents of Yakutia and Nizhny Novgorod (*p* = 0.0003) (Figure 6D).

### 3.4. Variations in General and Biochemical Blood Parameters in Relation to the Rate of Aging and Environmental Factors

Finally, we identified the blood parameters most strongly correlated with biological age acceleration (Supplementary Figure S3). The RDW-CV parameter exhibited the highest Pearson correlation coefficient (*p* = 0.7022), followed by: ALP (0.33), MCV (0.3), LYM (−0.285), MCHC (−0.28), WBC (0.24), creatinine (0.22), PLT (0.18), glucose (0.18), albumin (−0.16), HCT (0.15), MCH (−0.13), CRP (0.12), HGB (−0.11), and RBC (0.01).

Analysis of the constructed heat maps (Supplementary Figure S4) revealed patterns characteristic of different aging rates across the studied places of residence. In Yakutia, accelerated aging was primarily associated with elevated levels of RDW-CV, MCV, and ALP, while delayed aging was linked to reduced levels of RDW-CV, WBC, creatinine, PLT, and albumin. Furthermore, both accelerated and delayed aging were associated with higher HGB levels. Among residents of the metropolis of Nizhny Novgorod, no specific patterns were identified for individuals with negative age acceleration, other than a direct correlation between biological age acceleration and RDW-CV. In this group, accelerated aging was associated with elevated WBC, creatinine, and glucose levels. Residents of small towns in the Nizhny Novgorod region generally exhibited lower levels across all studied blood parameters. However, with increasing aging rate, a slight increase was noted in RDW-CV, WBC, MCH, and CRP levels. In the industrial city of Dzerzhinsk, MCH and MCHC levels remained elevated irrespective of the aging rate. Notably, accelerated aging in this group was associated with an increase in RDW-CV and a decrease in MCV, HGB, HCT, and albumin.

## 4. Discussion

Although chronological age is arguably the strongest risk factor for age-related diseases and mortality, it is crucial to distinguish it from biological aging [20,21,22,23]. Individuals of the same chronological age may exhibit vastly different susceptibilities to age-related diseases and death. This likely reflects differences in their underlying biological aging processes. Human life expectancy varies significantly under the combined influence of environmental factors, collectively known as the exposome [3,4,24]. Centenarians can be found across all population groups and geographical regions, even in extreme climatic conditions [25,26]. This suggests the existence of mechanisms that not only facilitate physiological adaptation to environmental factors but also contribute to extended longevity. Such biomarkers of aging will be crucial to enable evaluation of interventions aimed at promoting healthier aging.

In this study, we analyzed hematological and biochemical blood parameters of residents from an extreme cold climate (the Republic of Sakha (Yakutia)) and from the milder climate of Central Russia to identify aging-associated markers linked to environmental exposures. The study participants from Central Russia of the Nizhny Novgorod region were grouped according to the degree of urbanization, including a metropolis (Nizhny Novgorod), an industrial city (Dzerzhinsk), and two small towns with populations of less than 50,000 people (Semenov and Pavlovo). We identified specific biomarkers—namely albumin levels and RDW-CV—and distinct patterns for each studied climatic zone and urban environment that underscore the role of the exposome in aging processes.

Application of the PhenoAge model allows us to quantify biological age and to examine its associations with physiological and clinical parameters. Developed by Levine et al. [16], the PhenoAge model is a composite biological age estimator based on a weighted linear combination of chronological age and nine clinical biomarkers (see Section 2.4) that reflect key physiological systems. The PhenoAge is widely used in aging research and clinical studies [27,28,29,30] because it captures the cumulative physiological burden and overall health status, enabling early identification of accelerated aging phenotypes and informing potential interventions. In our previous work [31], we demonstrated that the PhenoAge model is suitable for estimating age for European ancestry and fully comparable with data obtained using established epigenetic clocks (e.g., Hannum DNAm age [32], Horvath DNAm age [33], and GrimAge [34]).

In this study, the biological age of Yakutia residents was found to be higher than their chronological age. This aligns with our prior epigenetic evidence of accelerated aging in the Yakut population, linked to region-specific differentially methylated regions that connect climate adaptation and aging [15]. Here, we identified age-associated physiological differences in Yakutia residents. These include higher levels of HCT, RDW-CV, ALP, and MPV, alongside a decline in albumin concentration with age. Serum albumin levels have been reported to be higher in populations from warmer, tropical environments [35]. This phenomenon may represent a biologically advantageous adaptation to heat stress. It also raises the possibility of genetic regulation of serum albumin levels, although the underlying mechanisms remain poorly understood [35]. Currently, there is no literature specifically addressing serum albumin levels in cold-adapted populations. However, it can be hypothesized that the observed decrease in serum albumin in northern populations is linked to higher basal metabolic demands. This link persists despite a diet rich in animal protein, as seen in Yakutia [36,37,38].

In contrast, the residents of the metropolis of Nizhny Novgorod and the industrial city of Dzerzhinsk exhibited approximately equal biological and chronological age on average. Although these cities impose a significant environmental burden (see city characteristics in Supplementary Materials, Sections S1.2.1 and S1.2.2), their milder climate compared to Yakutia’s appears to prevent average biological age from rising significantly above chronological age. This suggests that the impact of extreme climatic conditions makes a greater contribution to the development of age-related acceleration than high levels of urbanization [15,39]. Furthermore, the lifestyle and a combination of social factors, including quality of medical care, can also contribute to a broad range of health conditions and accelerated aging processes [30,40]. While this critical aspect was beyond the scope of our study, it represents an important direction for future research. Ultimately, the importance of urbanization—including environmental factors—is evident, as participants from environmentally sound small towns in the Nizhny Novgorod region showed a lower biological age in their early chronological years.

Although the machine learning-derived clusters did not show clear separation based on environmental conditions, certain distribution patterns were observed. One cluster was primarily from the Nizhny Novgorod region. Another cluster showed greater biological age acceleration and included participants from Yakutia and the metropolis of Nizhny Novgorod. The third cluster included participants from Yakutia and small towns. Comparisons of clusters revealed significant differences in aging rates, likely resulting from distinct aging-related patterns in blood parameters across different climatic zones.

Our analysis showed that RDW-CV was the key factor that consistently correlated positively with age acceleration across different climatic zones. The RDW-CV index reflects variability in red blood cell size, which changes with erythrocyte lifespan [41]. According to the literature data, an increased RDW-CV is a well-established risk factor for cardiovascular disease and cancer [42,43], while an increase in RDW with age is a known biological feature [44]. Given these findings, RDW may hold potential as a biomarker of biological aging [45]. The observed differences in hematological indices suggest that northern populations have distinct erythropoiesis patterns, with shorter erythrocyte lifespan and enhanced production, particularly in winter months [46]. Reduced erythrocyte longevity and increased hematopoiesis can act as compensatory mechanisms, allowing the hematopoietic system to adapt to the combined effects of extreme climatic, geographical, and ecological stressors [26]. However, these adaptations reduce the body’s physiological reserves, ultimately contributing to accelerated aging—a physiological cost of surviving in extreme northern environments. The role of the hematopoietic system in the aging process warrants further investigation.

Of interest are the data showing a region-specific pattern associated with accelerated aging. In Yakutia, accelerated aging was most strongly associated with increased MCV, ALP, and HGB concentrations. In the metropolis of Nizhny Novgorod, accelerated aging was accompanied by elevated WBC, creatinine, and glucose levels, while in the industrial city of Dzerzhinsk, it was marked by a decline in MCV, HGB, HCT, and albumin levels. Residents of small towns in the Nizhny Novgorod region were generally characterized by lower values across all studied blood parameters and exhibited slower aging rates. However, as aging accelerated, WBC, MCH, and CRP levels increased slightly.

### Study Limitations

We would also like to address the limitations of this study, which may have influenced the interpretation of the results.

A key limitation, common in biomedical research, is the relatively modest sample size (n = 445). However, we justify our sample sizes through a post hoc power analysis. The primary comparison (Yakutia, n = 153 vs. Nizhny Novgorod, n = 144) achieved 100% power at α = 0.05 (two-tailed) for the observed medium effect (d = 0.677). The smaller subgroups (Dzerzhinsk, n = 60; small towns, n = 88) also maintained over 80% power for medium-to-large effects.

The non-equivalent age distributions across the study cohorts, including differences in mean age, range, and proportions of younger versus older adults, may influence our results. Cohorts with younger mean ages may exhibit different biomarker profiles and PhenoAge estimates, potentially reflecting age-related physiological changes rather than climate-specific adaptation. Machine learning clustering may also preferentially identify age-related patterns, particularly if one cohort contains a substantially different age composition. Additionally, despite the PhenoAge being age-adjusted by design, residual differences in age distribution across cohorts might introduce systematic bias in biological age acceleration estimates.

The study cohorts had a significant imbalance in sex distribution, which may introduce bias in the overall PhenoAge estimates and machine learning clustering results. The proportion of female participants was higher than of male participants in all study groups, and we did not conduct a separate analysis of the effect of sex on the studied parameters.

Our study design lacked individual-level data on environmental exposures (e.g., temperature, air quality, altitude) and lifestyle confounders (e.g., socioeconomic status, diet, physical activity, smoking, alcohol consumption, medication use, and family history of longevity). We used regional residence as a proxy for environmental exposure, which prevented us from establishing direct causal links. The observed associations may be confounded by unmeasured regional factors. Future studies should address these limitations by conducting larger validation cohorts and incorporating emerging methodologies.

## 5. Conclusions

In this study, we applied the Levine PhenoAge model and machine learning to compare hematological and biochemical parameters from individuals to identify aging-associated markers influenced by environmental exposures. The cohort comprised residents of an extremely cold climate region and of several cities with a milder climate, representing varying levels of urbanization.

Our findings indicate that living in extreme climatic conditions, such as those in Yakutia, promotes age acceleration. This phenomenon appears to be related to changes in the intensity of protein metabolism, as evidenced by decreased blood albumin levels. Furthermore, a strong positive correlation between age acceleration and the functional parameter of erythrocytes (RDW-CV) was observed across different climatic zones. For inhabitants of the milder Central Russian climate, the residents of small towns exhibited the slowest age acceleration, highlighting urbanization as a driver of accelerated aging. Our findings broaden our understanding of the role of exposome in aging processes. Further detailed investigation of the identified biomarkers and patterns in a larger cohort could contribute to the development of a range of measures to promote healthy aging and population longevity, taking into account the impacts of climate and ongoing urbanization processes.

## Figures and Tables

**Table 1:** Hematological and biochemical parameters analyzed in the study.

Blood Tests	Reference Ranges	Recorded Values of the Studied Cohort (Mean ± SEM [Min–Max])
Hematological parameters		
Total number of red blood cells per liter (RBC), 10^12^/L	3.8–5.3	4.60 ± 0.03 [2.62–6.38]
Total number of white blood cells per liter (WBC), 10^9^/L	4.1–11.1	6.14 ± 0.09 [1.82–20.60]
Hemoglobin concentration (HGB), g/L	117–155	128.83 ± 0.77 [74.00–174.00]
Red blood cell volume (MCV), fL	83–100	89.28 ± 0.40 [79.30–115.60]
Hematocrit (HCT), %	35–45	41.17 ± 0.29 [23.00–58.80]
Mean corpuscular hemoglobin content (MCH), pg	32–37	28.13 ± 0.14 [16.20–39.60]
Mean corpuscular hemoglobin concentration (MCHC), g/L	318–342	315.22 ± 1.57 [245.00–423.00]
Percentage of lymphocytes (LYM), %	16–46	32.48 ± 0.39 [2.90–57.00]
Red blood cell distribution width by volume (RDW-CV), %	12.2–14.6	13.88 ± 0.07 [10.90–20.70]
Platelet count (PLT), 10^9^/L	150–400	265.05 ± 3.41 [81.00–654.00]
Biochemical parameters		
Albumin, g/L	35–50	44.08 ± 0.27 [29.50–77.00]
Glucose, mmol/L	3.3–5.6	4.66 ± 0.05 [2.59–11.62]
Creatinine, umol/L	44–80	90.03 ± 0.96 [38.50–213.00]
Alkaline phosphatase (ALP), U/L	35–130	169.25 ± 3.35 [0.00–776.00]
C-reactive protein (CRP), mg/L	0–5	6.24 ± 0.34 [0.00–8.70]

## Data Availability

The data used to support the findings of this study are available from the corresponding author upon request.

## Supplementary Materials

The following supporting information can be downloaded at: https://www.mdpi.com/article/10.3390/biology14121668/s1: Section S1. Ecological Characteristics of Studied Regions; Table S1: Comparison of the studied groups of residents by the rate of aging; Figure S1: Dependence of clinical and biochemical blood test parameters on chronological age in the study groups of residents. (A) Number of red blood cells per liter (RBC); (B) Hemoglobin concentration (HGB); (C) Mean corpuscular hemoglobin content (MCH); (D) Creatinine; (E) C-reactive protein (CRP); (F) Red blood cell volume (MCV); (G) Platelet count (PLT); (H) Alkaline phosphatase (ALP). The color lines mean Gaussian moving average of parameters in the study groups; Figure S2: Correlation matrix representing the correlations of principal components with general and biochemical blood parameters, age acceleration, chronological age, and biological age across the resident study groups; Figure S3: Correlation matrix of the correlation coefficients among general and biochemical blood test parameters, chronological age, biological age, and age acceleration in the resident study groups. Figure S4: Heat maps showing the relationship between blood parameters and age acceleration in the resident study groups. Columns, from left to right, represent: ALP, MCV, LYM, MCHC, WBC, creatinine, PLT, glucose, albumin, HCT, MCH, CRP, HGB, RBC, and age acceleration. Rows at the top show participants with the slowest biological aging (delayed aging), and rows at the bottom show those with the highest rate of aging. References [47,48,49,50,51,52,53,54,55,56] are cited in the Supplementary Materials.

## Abbreviations

The following abbreviations are used in this manuscript:

ALP	Alkaline phosphatase
CRP	C-reactive protein
HCT	Hematocrit
HGB	Hemoglobin
LYM	Lymphocytes
MCH	Mean corpuscular hemoglobin content
MCHC	Mean corpuscular hemoglobin concentration
MCV	Red blood cell volume
ML	Machine learning
PCA	Principal component analysis
PLT	Platelets
RBC	Red blood cells
RDW-CV	Red blood cell distribution width by volume
WBC	White blood cells
