# Supplementary material for: Age-Related Human Adaptation to Extreme Climatic Factors and Environmental Conditions
Source: Biology (Basel). 2025 Nov 25;14(12):1668. doi: 10.3390/biology14121668 (PMC12729927; doi:10.3390/biology14121668)
Supplement: Supplementary file 1 [file biology-14-01668-s001.zip › biology-3924081-supplementary.pdf]

Supplementary Materials

S1. Ecological Characteristics of Studied Regions

S1.1. Yakutia

The Republic of Sakha (Yakutia), located near the 62nd parallel north, is known as the northern pole of cold on Earth. Temperatures there can drop below -70°C, and winter lasts from October to April (according to National Centers for Environmental Information (NCEI) https://www.ncei.noaa.gov/).

The atmospheric air quality in the Republic of Sakha is generally assessed as satisfactory: the concentrations of harmful substances in the air do not exceed the established maximum permissible levels [1]. However, summer forest fires in Yakutia, along with emissions from transportation, significantly impact air quality [2].

Yakutia, with its vast network of water bodies, including more than 700,000 rivers and 825,000 lakes, faces significant challenges in ensuring access to clean and safe drinking water [3]. The primary sources of water pollution include the discharge of untreated wastewater, industrial and energy sector effluents, and waste from housing and communal services, as well as pollution from shipping activities [4]. Furthermore, the region's unique climatic conditions, characterized by an extended period of permafrost and limited thermal energy, reduce the natural self-purification capacity of soil and water sources [5].

S1.2. Central Russia region

S1.2.1 Nizhny Novgorod

Nizhny Novgorod, a major metropolis, serves as the administrative center of both the Nizhny Novgorod region and the Volga Federal District. The city occupies an area of 460 km^2 and has a population of 1,213,500 residents (according to the Federal State Statistics Service for the Nizhny Novgorod Region (2025)).

The Nizhny Novgorod region experiences a relatively milder climate with higher average temperatures and a shorter winter period. The average winter temperature in the Nizhny Novgorod region is -13°C. Geographically, the Nizhny Novgorod region is situated on the 56th parallel north latitude, aligning with cities such as Edinburgh (Scotland), the Alaska Peninsula (USA), and several large urban centers in Canada and Russia.

In 2021, the Nizhny Novgorod region ranked 11th among Russian regions in terms of vehicle fleet size, with 2.18% of the nation's registered vehicles. Motor vehicle emissions constitute a significant source of air pollution in Nizhny Novgorod, accounting for 75% of total atmospheric pollutants [6]. The sanitary condition of water sources in the Nizhny Novgorod region is considered satisfactory [7,8].

S1.2.2 Dzerzhinsk

Dzerzhinsk, a large industrial city, has a highly developed chemical industry, which accounts for 61.3% of its total production and serves as the primary source of air pollution. The main air pollutants in the Dzerzhinsk include ammonia, suspended particulate matter, formaldehyde, and nitrogen dioxide [9]. Dzerzhinsk is the second-largest and most industrially significant city in the Nizhny Novgorod region, with a population of 262,000 and a total area of 420 km^2.

The water quality of the Oka River in Dzerzhinsk is characterized by chronic pollution with organic compounds, copper, and nitrite nitrogen, all of which exceed established safety standards [8]. Periodically, phenol concentrations have reached levels 25 times the maximum permissible limit, posing a severe threat to public health. Additionally, the discharge of untreated wastewater has led to significant bacterial contamination in the Oka River. To mitigate this risk, chlorination is used in the drinking water supply. However, this treatment process can lead to the unintended formation of carcinogenic chlorinated organic compounds, further increasing health hazards [10].

S1.2.3. Small towns

S1.2.3.1 Semyonov

Semyonov is the administrative center of the Semyonovsky urban district. Semyonov covers an area of 25.9 km^2. As of January 1, 2022, its permanent population was 23,673 (according to the Federal State Statistics Service for the Nizhny Novgorod Region (2022)). There is no state air quality monitoring station directly within the city of Semyonov. The available air pollution data published in state reports (State Report "On the State of the Environment and Natural Resources of the Nizhny Novgorod Region" for 2009–2017) are derived from the nearest control point located in the city of Bor. The annual average concentrations of all monitored

pollutants were below established sanitary standards [1]. This is consistent with the general environmental conditions of the northern part of the Nizhny Novgorod region, an area characterized by a lack of major industrial activity. In both the Nizhny Novgorod region as a whole and the Semyonovsky district specifically, the leading causes of mortality are circulatory system diseases (68.4%), neoplasms (10.4%), digestive system diseases (3.6%), respiratory system diseases (1.2%), and infectious and parasitic diseases (1.1%) (according to official data published in https://semenov.nobl.ru/documents/active/139535/).

S1.2.3.2 Pavlovo

Pavlovo is a city specializing in mechanical engineering and metalworking. The city has a well-developed transport system due to its proximity to major transport routes and railways. Furthermore, its location in central European Russia provides fertile soils and favorable conditions for agriculture. According to the Federal State Statistics Service for the Nizhny Novgorod Region, the city's population was 49,307 at the beginning of 2025. Pavlovo covers a total area of approximately 39 km^2, which classifies it as a small city within the Nizhny Novgorod region. The main pollutants emitted into the atmosphere in Pavlovo include nitrogen dioxide and oxide, sulphur dioxide, carbon monoxide, benzene, toluene, and ethylbenzene (based on data from the "State of the Environment and Natural Resources of the Nizhny Novgorod Region", 2007-2024). Additionally, the use of mineral fertilizers and agrochemicals near water bodies contributes to the penetration of nitrates and phosphates into rivers and underground groundwater horizons, significantly contributing to the pollution of the Oka River [8].

References:

1. Resolution No. 165 Dated December 22, 2017 on the Approval of Hygienic Standards gn 2.1.6.3492-17 "Maximum Permissible Concentrations (MPC) of Pollutants in the Atmospheric Air of Urban and Rural Settlements". Available online: https://docs.cntd.ru/document/556185926 (accessed on 26 September 2025)
2. Chemezov, E.; Sosina, S. State of the atmosphere of the Republic of Sakha (Yakutia). *Symb. Sci.* **2017**, *1*. Available online: https://cyberleninka.ru/article/n/sostoyanie-atmosfery-respubliki-saha-yakutiya (accessed on 3 November 2023).
3. Nikolaeva, N. Geoecological Approach to Justification of Priority Directions for Reducing Negative Ecological Impacts during Implementation on the South Yakutia of Large Energy Projects in the Republic of Sakha (Yakutia). *Proc. IOP Conf. Ser. Earth Environ. Sci.* **2021**, *720*, 012016. https://doi.org/10.1088/1755-1315/720/1/012016.
4. Burtseva, T.; Uvarova, T.; Savvina, M.; Shadrin, V.; Avrusin, S.; Chasnyk, V. Health status of Native people living in the Republic of Sakha (Yakutia). *Int. J. Circumpolar Health* **2013**, *72*, 21166.
5. Savvinov, G.N.; Velichenko, V.V. Fuel and energy complex of Yakutia: Environmental aspects. *Proc. IOP Conf. Ser. Earth Environ. Sci.* **2021**, *808*, 012062. https://doi.org/10.1088/1755-1315/808/1/012062.
6. Bolshakova, A.D.; Zaznobina, N.I.; Kovaleva, T.A. The role of green spaces in the improvement of the urban population health quality (on the example of Nizhny Novgorod). *Samara J. Sci.* **2023**, *12*, 27–33.
7. Aladyshkina, A.; Lakshina, V.; Leonova, L.; Maksimov, A. Ecological and economic modeling of environmental influence on disease incidence in children examplified by Nizhny Novgorod region. *Sotsial'nye Aspekty Zdorov'ya Naseleniya* **2018**, *2*, 8.
8. State Report "On the Sanitary and Epidemiological Well-Being of the Population in the Nizhny Novgorod Region in 2024". Available online: https://52.rospotrebnadzor.ru (accessed on 26 September 2025)
9. Sotkina, S.A.; Badina, O.N.; Shevchenko, I.A.; Bikmaeva, A.V. The ecological condition of the city of Dzerzhinsk on the degree of soil pollution with heavy metals. *Adv. Curr. Nat. Sci.* **2017**, *6*, 96–101. (In Russian)
10. Georgiadi, A.; Sharapova, E.; Danilenko, A. Urban impact on water quality in the rivers of Central Russia. *Proc. IOP Conf. Ser. Earth Environ. Sci.* **2021**, *834*, 012030. https://doi.org/10.1088/1755-1315/834/1/012030.

Table S1. Comparison of the studied groups of residents by the rate of aging.

Aging rate	Yakutia		Nizhny Novgorod		Small towns		Dzerzhinsk	
	Number of people	%	Number of people	%	Number of people	%	Number of people	%
Normal	49	32	92	64	24	27	34	56
Delayed	36	24	31	22	39	44	18	30
Accelerated	68	44	21	15	25	28	8	13

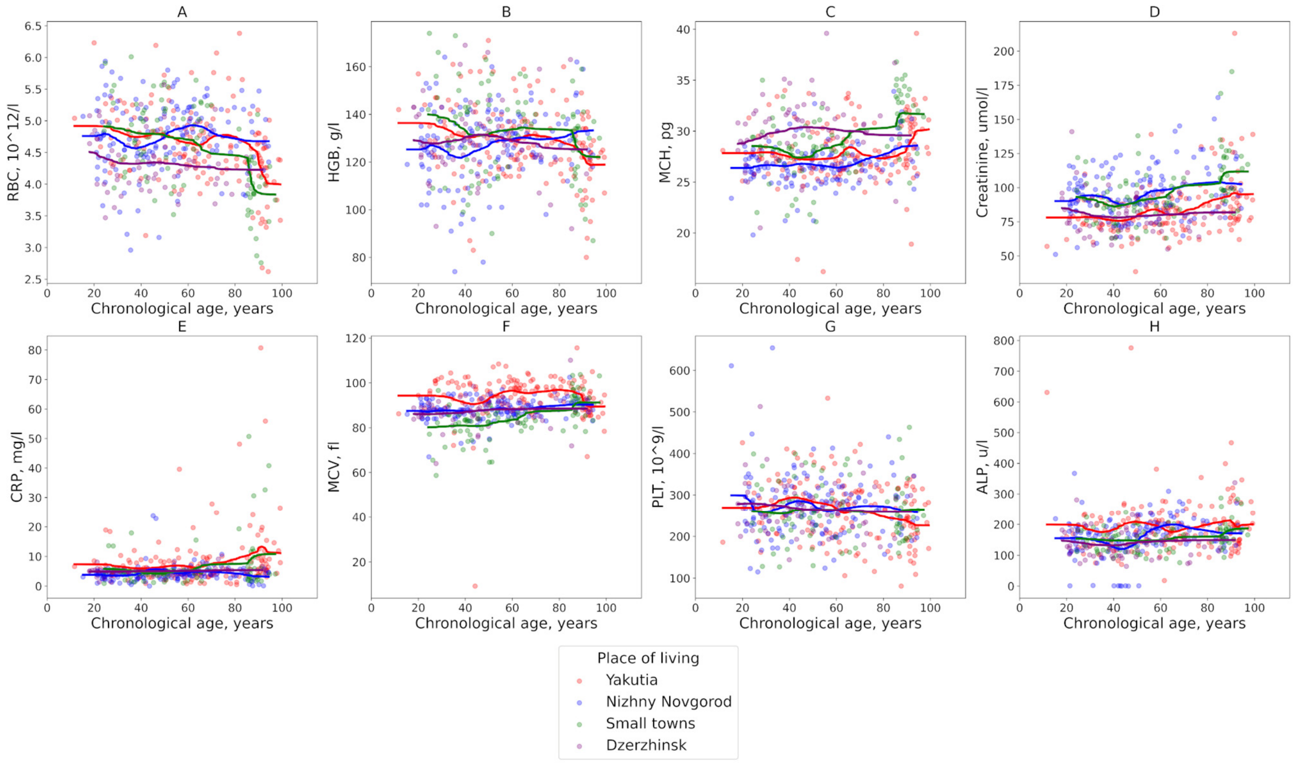

Figure S1 Dependence of clinical and biochemical blood test parameters on chronological age in the study groups of residents. (A) Number of red blood cells per liter (RBC); (B) Hemoglobin concentration (HGB); (C) Mean corpuscular hemoglobin content (MCH); (D) Creatinine; (E) C-reactive protein (CRP); (F) Red blood cell volume (MCV); (G) Platelet count (PLT); (H) Alkaline phosphatase (ALP). The color lines mean Gaussian moving average of parameters in the study groups.

The data revealed declines in RBC and HGB after 80 years of age in participants from Yakutia and the small towns of the Nizhny Novgorod region (Figure S1 A, B). MCH increased after 80 years in indigenous residents of Yakutia and after 50 years in participants from the small towns in the Nizhny Novgorod region (Figure S1 C). Creatinine levels increased after 50 years in all groups except Dzerzhinsk, with the highest average values observed in Yakutia (Figure S1 D). CRP levels began to rise after 60 years (Figure S1 E). Higher ALP levels were consistently observed throughout the lifespan in Yakutia, while PLT gradually decreased after 40 years in this group (Figure S1 G, H). Furthermore, MCV was higher in residents of Yakutia but declined sharply after 90 years. In contrast, residents of the small towns of the Nizhny Novgorod region had lower MCV values, which gradually increased after 50 years (Figure S1 F).

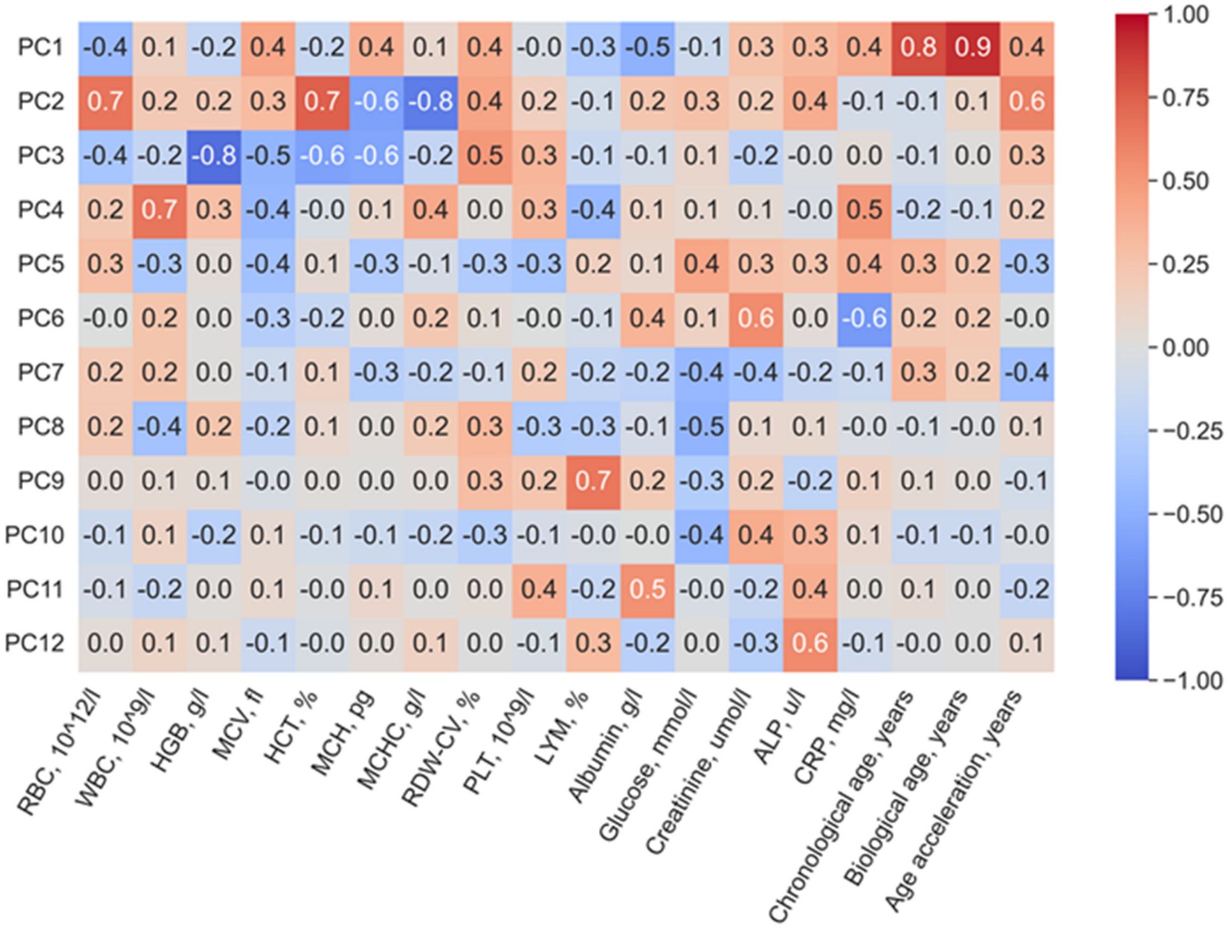

Figure S2. Correlation matrix representing the correlations of principal components with general and biochemical blood parameters, age acceleration, chronological age, and biological age across the resident study groups.

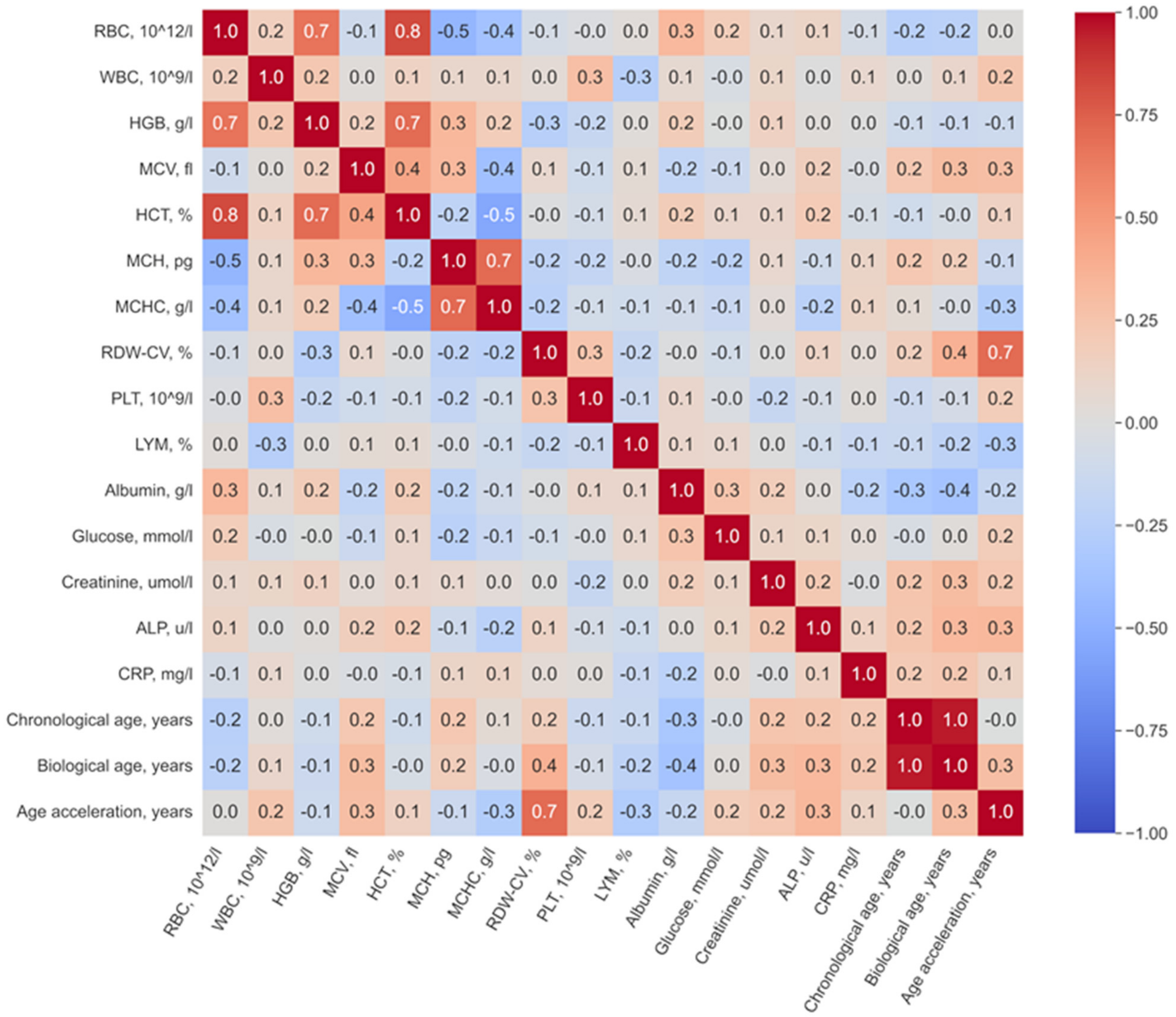

Figure S3. Correlation matrix of the correlation coefficients among general and biochemical blood test parameters, chronological age, biological age, and age acceleration in the resident study groups.

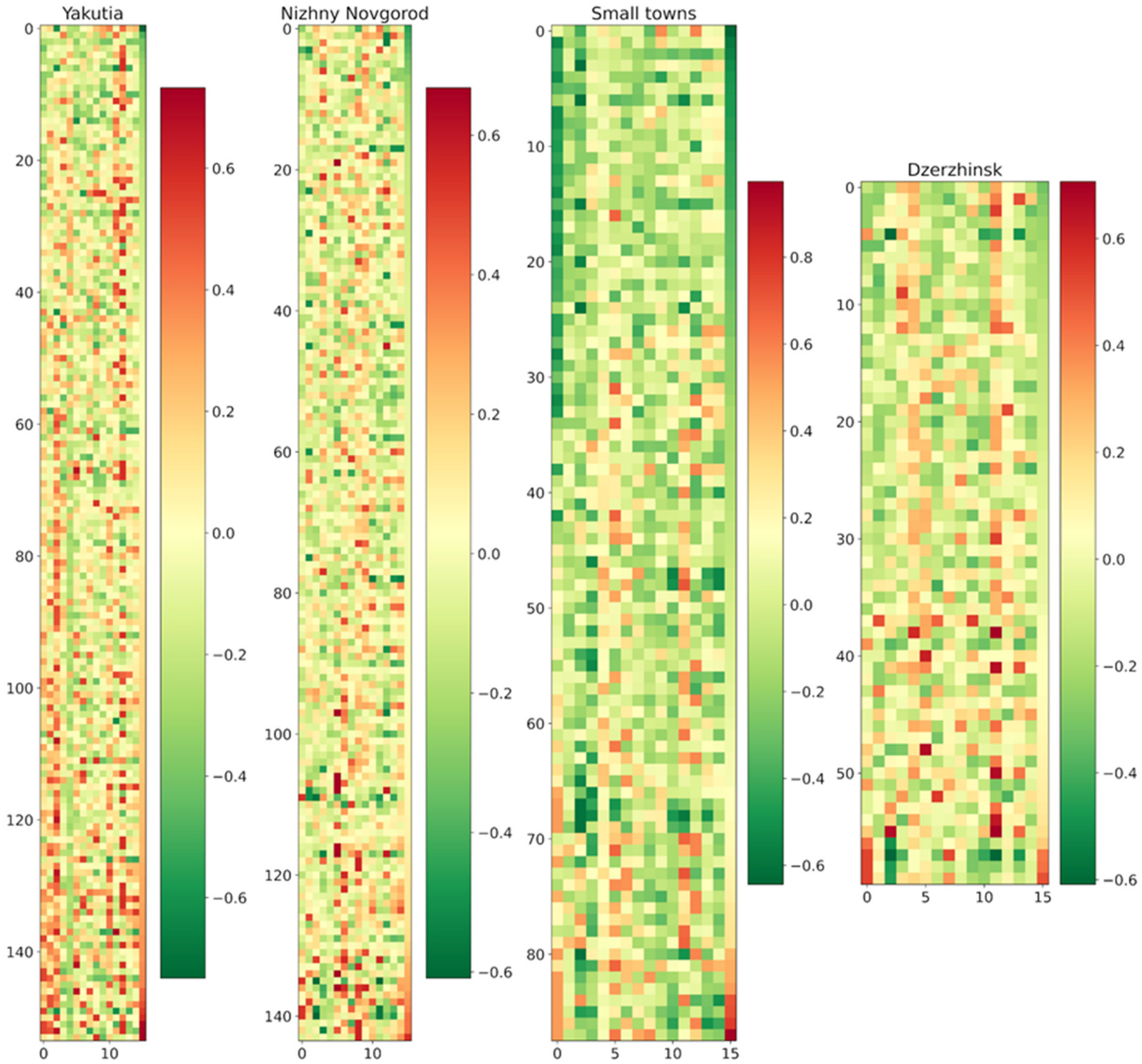

Figure S4. Heat maps showing the relationship between blood parameters and age acceleration in the resident study groups. Columns, from left to right, represent: ALP, MCV, LYM, MCHC, WBC, creatinine, PLT, glucose, albumin, HCT, MCH, CRP, HGB, RBC, and age acceleration. Rows at the top show participants with the slowest biological aging (delayed aging), and rows at the bottom show those with the highest rate of aging.
